# Supplementary material for: HIVprotI: an integrated web based platform for prediction and design of HIV proteins inhibitors
Source: J Cheminform. 2018 Mar 9;10:12. doi: 10.1186/s13321-018-0266-y (PMC5845081; doi:10.1186/s13321-018-0266-y)
Supplement: Supplementary file 2 — Additional file 2. Source code of HIVProtI web server. [file 13321_2018_266_MOESM2_ESM.zip › HIVprotI_Source-code/hivprotI/weka-3-6-6/doc/weka/experiment/ResultMatrixSignificance.html]

ResultMatrixSignificance


---


|  |  |  |
| --- | --- | --- |
| **Overview** \| **Package** \| **Class** \| **Tree** \| **Deprecated** \| **Index** \| **Help** \| **Weka's home** | |  |
| **PREV CLASS**   **NEXT CLASS** | **FRAMES**    **NO FRAMES**     **All Classes** |  |
| SUMMARY: NESTED \| FIELD \| CONSTR \| METHOD | DETAIL: FIELD \| CONSTR \| METHOD |  |


---


## weka.experiment Class ResultMatrixSignificance

```
java.lang.Object
  weka.experiment.ResultMatrix
      weka.experiment.ResultMatrixSignificance
```

**All Implemented Interfaces:**: java.io.Serializable, RevisionHandler

---

``` public class ResultMatrixSignificance extends ResultMatrix ```

This matrix is a container for the datasets and classifier setups and
their statistics. It outputs only the significance indicators - sometimes
good for recognizing patterns.

**Version:**
:   $Revision: 1.5 $

**Author:**
:   FracPete (fracpete at waikato dot ac dot nz)

**See Also:**: Serialized Form

---

**Field Summary**

**Fields inherited from class weka.experiment.ResultMatrix**

`LEFT_PARENTHESES, LOSS_STRING, RIGHT_PARENTHESES, SIGNIFICANCE_LOSS, SIGNIFICANCE_TIE, SIGNIFICANCE_WIN, TIE_STRING, WIN_STRING`


**Constructor Summary**

`ResultMatrixSignificance()`
  
          initializes the matrix as 1x1 matrix

`ResultMatrixSignificance(int cols,
int rows)`
  
          initializes the matrix with the given dimensions

`ResultMatrixSignificance(ResultMatrix matrix)`
  
          initializes the matrix with the values from the given matrix


| **Method Summary** | |
| --- | --- |
| `void` | `clear()`             removes the stored data but retains the dimensions of the matrix |
| `java.lang.String` | `getDisplayName()`             returns the name of the output format |
| `java.lang.String` | `getRevision()`             Returns the revision string. |
| `static void` | `main(java.lang.String[] args)`             for testing only |
| `void` | `setShowStdDev(boolean show)`             sets whether to display the std deviations or not - always false! |
| `java.lang.String` | `toStringHeader()`             returns the header of the matrix as a string |
| `java.lang.String` | `toStringKey()`             returns returns a key for all the col names, for better readability if the names got cut off |
| `java.lang.String` | `toStringMatrix()`             returns the matrix as plain text |
| `java.lang.String` | `toStringRanking()`             returns the ranking in a string representation |
| `java.lang.String` | `toStringSummary()`             returns the summary as string |

**Methods inherited from class weka.experiment.ResultMatrix**

`addHeader, assign, clearHeader, clearRanking, clearSummary, getAverage, getColCount, getColHidden, getColName, getColNameWidth, getColOrder, getCount, getCountWidth, getDisplayCol, getDisplayRow, getEnumerateColNames, getEnumerateRowNames, getHeader, getMean, getMeanPrec, getMeanWidth, getPrintColNames, getPrintRowNames, getRemoveFilterName, getRowCount, getRowHidden, getRowName, getRowNameWidth, getRowOrder, getShowAverage, getShowStdDev, getSignificance, getSignificanceCount, getSignificanceWidth, getStdDev, getStdDevPrec, getStdDevWidth, getVisibleColCount, getVisibleRowCount, headerKeys, setColHidden, setColName, setColNameWidth, setColOrder, setCount, setCountWidth, setEnumerateColNames, setEnumerateRowNames, setMean, setMeanPrec, setMeanWidth, setPrintColNames, setPrintRowNames, setRanking, setRemoveFilterName, setRowHidden, setRowName, setRowNameWidth, setRowOrder, setShowAverage, setSignificance, setSignificanceWidth, setSize, setStdDev, setStdDevPrec, setStdDevWidth, setSummary, toString`

**Methods inherited from class java.lang.Object**

`equals, getClass, hashCode, notify, notifyAll, wait, wait, wait`

**Constructor Detail**

### ResultMatrixSignificance

```
public ResultMatrixSignificance()
```

:   initializes the matrix as 1x1 matrix

---


### ResultMatrixSignificance

```
public ResultMatrixSignificance(int cols,
                                int rows)
```

:   initializes the matrix with the given dimensions

---


### ResultMatrixSignificance

```
public ResultMatrixSignificance(ResultMatrix matrix)
```

:   initializes the matrix with the values from the given matrix

    **Parameters:**: `matrix` - the matrix to get the values from


**Method Detail**

### getDisplayName

```
public java.lang.String getDisplayName()
```

:   returns the name of the output format

    :   **Specified by:**: `getDisplayName` in class `ResultMatrix`

---


### clear

```
public void clear()
```

:   removes the stored data but retains the dimensions of the matrix

    :   **Overrides:**: `clear` in class `ResultMatrix`

---


### setShowStdDev

```
public void setShowStdDev(boolean show)
```

:   sets whether to display the std deviations or not - always false!

    :   **Overrides:**: `setShowStdDev` in class `ResultMatrix`

---


### toStringMatrix

```
public java.lang.String toStringMatrix()
```

:   returns the matrix as plain text

    :   **Specified by:**: `toStringMatrix` in class `ResultMatrix`

---


### toStringHeader

```
public java.lang.String toStringHeader()
```

:   returns the header of the matrix as a string

    :   **Specified by:**: `toStringHeader` in class `ResultMatrix`
    :   **See Also:**: `ResultMatrix.m_HeaderKeys`, `ResultMatrix.m_HeaderValues`

---


### toStringKey

```
public java.lang.String toStringKey()
```

:   returns returns a key for all the col names, for better readability if
    the names got cut off

    :   **Specified by:**: `toStringKey` in class `ResultMatrix`

---


### toStringSummary

```
public java.lang.String toStringSummary()
```

:   returns the summary as string

    :   **Specified by:**: `toStringSummary` in class `ResultMatrix`

---


### toStringRanking

```
public java.lang.String toStringRanking()
```

:   returns the ranking in a string representation

    :   **Specified by:**: `toStringRanking` in class `ResultMatrix`

---


### getRevision

```
public java.lang.String getRevision()
```

:   Returns the revision string.

    :   **Returns:**: the revision

---


### main

```
public static void main(java.lang.String[] args)
```

:   for testing only


---


|  |  |  |
| --- | --- | --- |
| **Overview** \| **Package** \| **Class** \| **Tree** \| **Deprecated** \| **Index** \| **Help** \| **Weka's home** | |  |
| **PREV CLASS**   **NEXT CLASS** | **FRAMES**    **NO FRAMES**     **All Classes** |  |
| SUMMARY: NESTED \| FIELD \| CONSTR \| METHOD | DETAIL: FIELD \| CONSTR \| METHOD |  |


---
